# Mid-term implant survival, functional and radiological results and mechanical complications of mega-prosthetic reconstruction around the knee with *the PENTA*® system

**DOI:** 10.1007/s00402-021-04108-3

**Published:** 2021-08-21

**Authors:** Harzem Özger, Bugra Alpan, Ahmet Salduz, Volkan Gurkan, Mustafa Sungur, Natig Valiyev, Levent Eralp

**Affiliations:** 1 Istanbul Faculty of Medicine, Department of Orthopaedics and Traumatology (Retired), Istanbul University, Capa 34093 Fatih, Istanbul Turkey; 2 School of Medicine, Department of Orthopaedics and Traumatology, Acibadem Mehmet Ali Aydinlar University, Kayısdagi, Cad. 34752 Atasehir, Istanbul Turkey; 3 Medical Faculty, Department of Orthopaedics and Traumatology, Bezm-i Alem Vakif University, Fatih, Istanbul Turkey; 4 Department of Orthopaedics and Traumatology, AcibademAtakent Hospital, Turgut Ozal Bulvari No:16, 34303 Kucukcekmece, Istanbul Turkey; 5 Department of Orthopaedics and Traumatology, Acibadem Maslak Hospital, Buyukdere Caddesi No:40, 34457 Sariyer, Istanbul Turkey; 6 Istanbul Faculty of Medicine, Department of Orthopaedics and Traumatology , Istanbul University, Capa 34093 Fatih, Istanbul Turkey

**Keywords:** Limb salvage, Bone neoplasms, Surgical oncology, Knee prosthesis, Reconstructive surgical procedure, Prosthesis failure, Prosthesis survival, Knee joint

## Abstract

**Aim:**

Mega-prosthetic reconstruction is the most common treatment method for massive osteoarticular defects caused by tumor resection around the knee. The new implant is a highly modular rotational-hinged megaprosthesis system with a distinct pentagonal stem geometry and variable implantation options. The aim of this study is to present the mid-term implant survival characteristics, functional and radiological results and mechanical complication profile of the new megaprosthesis.

**Methods:**

One hundred and one mega-prosthetic knee reconstruction procedures in 90 patients (M/F: 51/39) utilizing the new implant system were retrospectively analyzed. In 68 patients, the megaprosthesis was used for primary reconstruction following tumor resection while it was used for revision of other implants in 22. The mean age was 28.5 (7–66) years and the mean follow-up was 59.2 (24–124) months. The most common primary pathology was osteosarcoma with 63–70% patients, the most common anatomical site of involvement was the distal femur with 56–62% patients.

**Results:**

Henderson Type 2 failure (aseptic loosening) was seen in only 2–2.2% patients while Type 3 (structural failure) was seen in 29–32.2% Although the 5-year anchorage survival rate was 94.3%, overall mechanical implant survival was 76.1% at 5 years due to a relatively high failure rate in the first-generation hinge mechanism of the implant. The 5-year hinge survival rate demonstrated a significant improvement rate from 61.7% to 87.2% between the first and second generations of the implant (*p* = 0.027). The mean MSTS score was 24 out of 30 (14–29). The mean cumulative ISOLS radiographic score for index megaprosthesis operations was 19.7 (12–24), which corresponded to excellent outcome.

**Conclusion:**

The new megaprosthesis system is a reliable choice for the reconstruction of tumor-related massive osteoarticular defects around the knee. Although long-term follow-up is necessary for a definitive evaluation of the implant's survival characteristics, midterm follow-up yields exceptional anchorage properties related to pentagonal stem geometry with very good functional outcomes.

## Introduction

While advances in imaging modalities, oncologic treatments and surgical technique have set the ground for limb salvage surgery to become the mainstay of management in musculoskeletal tumors over the last four decades, improved implant design has been particularly instrumental in providing better quality of life to limb salvage patients. Modular mega-prosthetic implants are the most common means of non-biological reconstruction around the knee joint following tumor resection. While these implants facilitate early weight-bearing and rapid restoration of function [1–5], the complex biomechanics of the knee, the loss of static and dynamic periarticular soft tissue stabilizers due to tumor resection and the high-demand use of the limb associated with young age contribute to mega-prosthetic failure through various non-oncological mechanisms with longer follow-up [1, 4–7]. The design and manufacturing qualities of a mega-prosthetic knee implant as well as the surgical technique directly impact the longevity of reconstruction and the quality of life.

Taking into consideration the disadvantageous aspects of previous locally manufactured implants and national difficulties in import issues, PENTA modular extremity reconstruction system was co-developed by the senior author (HO) and TIPSAN (Izmir, Turkey) orthopedic device company. Minimizing mega-prosthetic failure requiring major revision and maximizing limb function were the design goals. The implant was named after the pentagonal cross section of its stem and was made available for the local market in 2009 with CE certification. The implant's hinge design was modified in 2011 and the 2nd-generation hinge mechanism was used from 2012 onwards.

This study aims to evaluate the mid-term implant survival rate, functional results, radiological results and the mechanical complications of mega-prosthetic reconstruction around the knee with PENTA® system.

## Patients and methods

### Study population

The study was conducted as a retrospective analysis based on the tumor registries of 3 different orthopedic oncology centers following approval by institutional review board. A total of 188 consecutive patients were found to have undergone megaprosthesis reconstruction, including all anatomic locations (knee, hip, shoulder) between 2009 and 2020, during which PENTA system was used exclusively for mega-prosthetic reconstruction in all unless a partial revision of a different implant was being performed. Exclusion of patients with less than 2 years of follow-up and/or operated for non-neoplastic conditions yielded 101 PENTA mega-prosthetic knee reconstruction procedures in 90 (M/F: 51/39) patients. Provided having a minimum 2-year follow-up, 11 additional surgeries performed in 10 out of these 90 patients due to structural PENTA failure were included in the study (Table 1). Operations were performed by 4 different surgeons; 49–54% patients were operated by the senior author (HO). While 80–89% patients had a malignant primary pathology, the most common primary pathology was osteosarcoma with 63–70% patients. The primary tumor was located in the distal femur in 56–62% patients and proximal tibia in 34–38%. In 68–76% patients, PENTA was used for primary reconstruction following tumor resection while it was used for revision of other implants in 22–24%. Twelve out of these 22 patients underwent revision for aseptic loosening, 5 for structural failure and 5 for chronic deep infection. PENTA was implanted in the second stage of revision in the infection cases. The 1st-generation hinge design was used in the index PENTA procedure in 28–31% patients, while the 2nd-generation hinge was used in index surgery of 62–69% patients. The mean age was 28.5 (7–66) years at the time of index PENTA surgery and the mean follow-up was 59.2 (24–124) months. Fifty-four (60%) patients received oncological (chemotherapy, radiotherapy or both) treatment peri-operatively with regard to the index PENTA surgery. All patients underwent routine staging and follow-up procedures pre- and postoperatively (eg. plain radiography, MRI, CT chest, PET/CT or whole-body MRI).

**Table 1:** Demographic features of the patients

Number of patients	90
Number of surgeries	101
Mean age (years)	28.5 (7–66)
Mean follow-up (months)	59.2 (24–124)
*Primary diagnosis of the patients*	
Osteosarcoma	63 (70.0%)
Chondrosarcoma	6 (6.7%)
Ewing's Sarcoma	7 (3.3%)
Other malignant (PS, FS, SS, lymphoma, met Ca)	8 (8.9%)
Benign aggressive (GCTB, DF, ChB, ABC)	10 (11.1%)
*Localization of primary tumor*	
Distal femur	56 (62.2%)
Proximal tibia	34 (37.8%)
*Setting of PENTA surgery*	
Primary implant following tumor resection	68 (76%) pts
Revision implant	22 (24%) pts

*PS* pleomorphic sarcoma, *FS* fibrosarcoma, *SS* synovial sarcoma, *met Ca* metastatic carcinoma, *GCTB* giant cell tumor of bone, *DF* desmoplastic fibroma, *ChB* chondroblastoma, *ABC* aneurysmal bone cyst, *pts *patients

### PENTA modular extremity reconstruction system

PENTA is a set of implants designed to reconstruct irreparable defects involving the hip, knee, shoulder and elbow joints caused by tumor resection and revision of megaprostheses. All metallic components of the implant are manufactured from Ti6Al4V except for the femoral component for proximal tibia resection prosthesis, which is made of CoCrMo. The inserts and bushings in the joint mechanism are made of ultra-high molecular weight polyethylene (UHMW-PE) [8]. The design rationale and main advantages of the implant system can be described in relation to its anchorage, modularity and articulation characteristics.

*Anchorage* The name of the implant is derived from the pentagonal cross-section of its stem. The stem is slightly tapered towards the tip to prevent both unnecessary bone loss and stress shielding. Although the stem design particularly favors cement-less implantation, both hydroxyapatite (HA)-coated and rough-sanded stem options are available for cement-less and cemented implantation. The pentagonal stem geometry is aimed at combining the advantages of square and hexagonal cross-sectional stems, which are increased primary rotational stability and decreased risk of bone damage during cement-less implantation, respectively. Furthermore, HA-coating improves primary stability against pull-out forces by increasing friction at the bone-implant interface and ensures secondary stability through osseo-integration. A wide array of stem options are present, in terms of stem length (120–150–200 mm) stem diameter (12–22 mm) and stem curvature (straight or anatomical), to guarantee the best anchorage in all intraoperative scenarios. All stems have collars to prevent subsidence. The stem collars are also HA-coated to promote extracortical bone bridging and thus better secondary stability.

*Modularity* PENTA megaprosthesis system has a highly modular design to allow fine-tuning of the reconstruction in terms of limb length and rotational alignment. The limb length can be adjusted at 1-cm intervals with modular extension components. On the other hand, connections between the modular parts are secured with 3 different features; toothed-connection between all parts, conical press-fit connection between the parts and axial trans-fixation bolt, which spans the length of all components on each side of the joint. Distal femoral bodies for distal femoral resection and femoral components for proximal tibial resection have 5° of valgus. On the other hand, proximal tibial bodies for proximal tibial resection and the tibial baseplates for distal femoral resection are built without laterality. Size options are available for these modular parts.

**Fig. 1:**
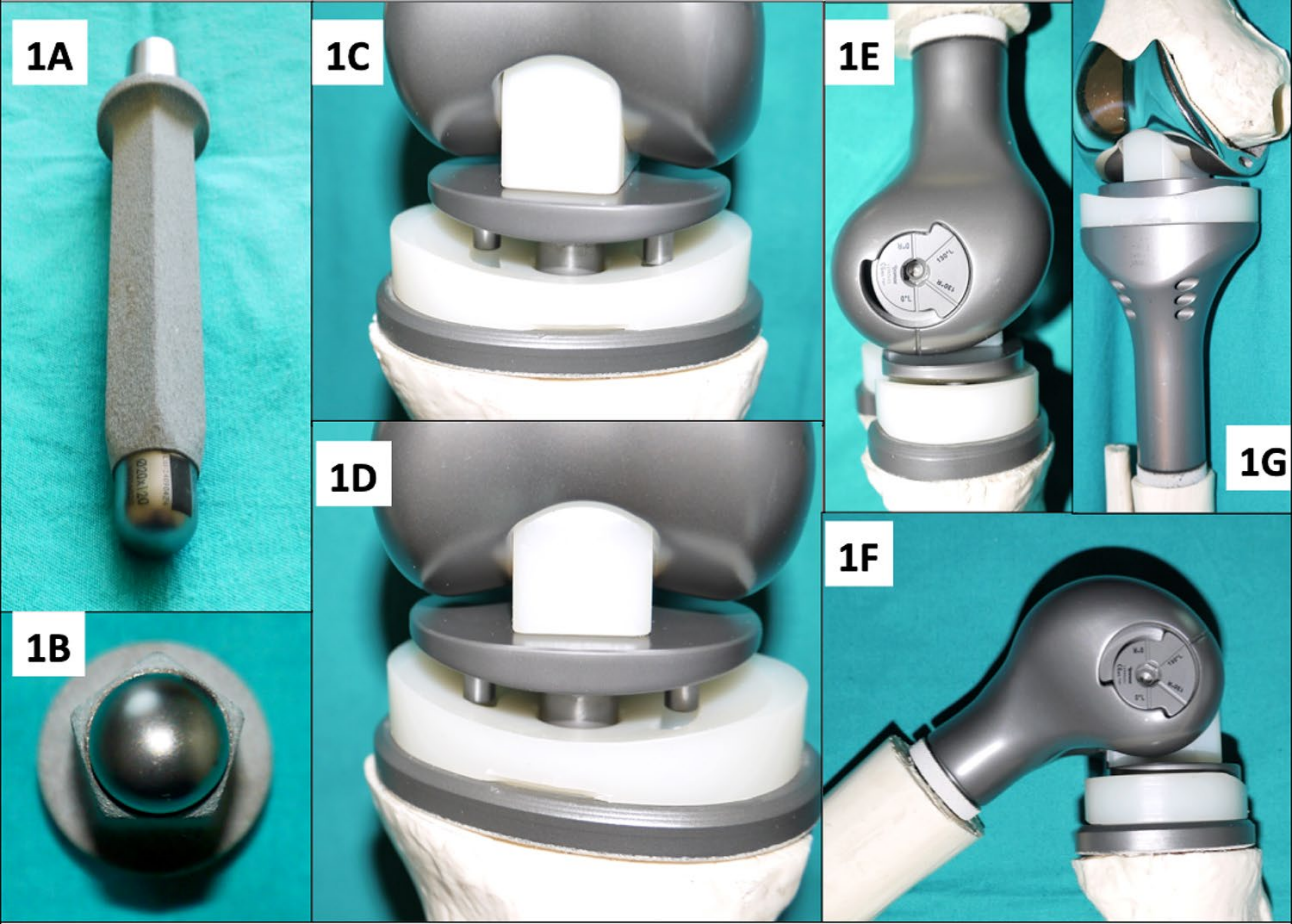
Design features of the PENTA® are shown. **A** and **B** Demonstrate the pentagonal, hydroxyapatite-coated stem. **C** and **D** Demonstrate the rotational ability in both directions and the curbed-stop mechanism with the hinge distracted. **E** and **F** Demonstrate the extension and flexion blockage by the stepped rotation blocks on the axle head and inside the axle socket of the femoral component. **G** Demonstrates the proximal tibia body articulating with the femoral component

*Articulation* PENTA knee megaprosthesis has a rotational hinge mechanism, which connects the femoral and tibial components through a yoke assembly (tibial rotation piece). The hinge formed by the distal femur and the yoke allows a 0°–130° range of motion in the sagittal plane. While hyperextension was solely blocked by a thick bumper insert in the first-generation hinge mechanism, the design was modified and a second-generation hinge was used from 2012 onwards. The 2nd-generation hinge, which is still currently in use, limits maximum flexion and extension by corresponding stepped rotation blocks on the axle head and inside the axle socket of the femoral component. The sharp edge of the intercondylar notch, which came into contact with the bumper insert during hyperextension, was also rounded off in the 2nd-generation hinge. The yoke can rotate 15° both internally and externally through its articulation with the proximal tibia. Rotation occurs around the cylindrical yoke post, which resides unconstrained in the proximal tibia. However, rotation is actually limited by 2 features. The yoke has a convex undersurface with 2 projections while the concave upper surface of the tibial insert, which is fixed to the proximal tibia, has 2 grooves corresponding to the projections under the yoke. The interface geometry of these two non-spherical components ensures a smooth stop at the endpoints of rotation, where the under-projections are limited by the grooves (Fig. 1).

### Postoperative rehabilitation and follow-up

Isometric quadriceps exercises were started as soon as adequate analgesia was achieved. Postoperatively, prophylactic intravenous antibiotics were used in all cases until the removal of all drains. The drains were removed at an average of 4 days. Primary cases were allowed full weight-bearing immediately after surgery with two crutches. However, weight-bearing was protracted up to 6 months in revision cases with graft impaction. Following proximal tibial resections, knee immobilizers were used for 6 weeks to allow for healing of the extensor mechanism. Knee flexion was allowed after 6 weeks with the goal to obtain 15° of flexion for every two weeks. Patients were followed up every three months for the first 2 years, every 6 months for the next three years and annually after 5 years.

### Evaluation methods

Endo-prosthetic reconstruction failures were classified according to failure modes described by Henderson et al. [9]. Any soft tissue complication requiring invasive procedures ranging from hematoma aspiration to flap reconstruction was accepted as Type 1 failure. Prosthetic stems with radiographically and clinically shown macro-motion in the absence of septic clinical, radiological and laboratory findings were accepted as having Type 2 failure. Clinical pathological movement and/or any radiographic finding of loss of structural integrity were accepted as Type 3 failure. Patients with chronic pain in anchorage sites regardless of radiographic appearance or with stem loosening in the presence of laboratory findings, with peri-prosthetic abscess formation or chronic fistula and with culture-positive peri-prosthetic effusions were accepted as having Type 4 failure. Patients with clinically and/or radiologically detected and biopsy proven lesions in the vicinity of megaprosthesis were accepted as having Type 5 failure. All 5 types of failure were screened for and both mechanical and non-mechanical failures were reported for a comprehensive overview of the study population. However, the outcome analysis focused on mechanical (Type 2 and 3) failures, in conformity with the aim of the study. Mechanical failures were established with clinical and radiological findings. Survival analysis of the implant was done with regard to overall mechanical survival, anchorage survival and hinge mechanism survival. Anchorage failure was designated as any breakage or aseptic loosening of the anchorage components (including stem, tibial baseplate of the distal femoral replacement or femoral component of proximal tibial replacement). Hinge failure was designated as any breakage of the hinge mechanism components (including bumper insert, axle, axle bushing, tibial rotation piece, tibial insert). Any revision surgery, which had already been performed or was being planned at the time of data collection, was considered as the end-point of survival for both anchorage and hinge components. For patients with multiple modes of mechanical failure, the overall mechanical survival was determined according to whichever failure developed first.

Radiological outcomes were assessed according to the ISOLS radiographic scoring system for implants (bone remodeling, interface, anchorage, implant body, implant articulation, extracortical bone bridging) [10]. Each radiographic parameter was scored as 1 (poor), 2 (fair), 3 (good) or 4 (excellent). The radiographic scores were used both individually to objectively identify specific failure types (Type 2; Type 3—hinge failure, anchorage breakage, peri-prosthetic fracture) and also collectively to give an overall score for the implant. Peri-prosthetic fractures were also assessed according to the Unified Classification System (UCS), which provides a better understanding of the relationship of the implant to the fracture.

Functional outcomes were evaluated using Musculoskeletal Tumor Society (MSTS) Scoring System [11].

Statistical analysis was done using SPSS 20 (IBM). Descriptive statistics of the patients, survival of the implants, and the association of various clinical variables and failure of the implants were analyzed using Kaplan–Meier survivorship curve, log-rank test and Cox proportional hazards regression model.

## Results

### Functional outcome

The mean MSTS was 23.8 (14–29) out of 30 including all patients who underwent index PENTA procedure. MSTS scores were significantly better after primary PENTA procedures (24.6 ± 2.9) compared to revision PENTA procedures (21.4 ± 3.7) (*p* = 0.001). MSTS scores were also significantly better after distal femoral reconstructions (24.6 ± 3.3) compared to proximal tibia reconstructions (22.5 ± 3.2) (*p* = 0.003). The mean MSTS score for patients who underwent primary PENTA procedure with the 2nd-generation implant was 24.7 ± 3.2. Seventy-eight patients with good to excellent radiographic interface scores had a mean MSTS score of 24.2 ± 3.2, which was significantly higher when compared to the mean score of 21.7 ± 3.8 for 12 patients with poor to fair interface scores according to paired samples test (*p* = 0.000). MSTS scores did not correlate with any other demographic parameter, mechanical mode of failure, generation of implant (1st vs. 2nd) or overall ISOLS radiographic score. Although clinical symptoms of knee discomfort were resolved after revision of failed hinge mechanisms, MSTS scores did not demonstrate a significant change.

### Radiological outcome

The mean overall ISOLS radiographic implant score was 19.7 ± 2.9 out of 24 for all patients following the index PENTA operation. The mean overall radiographic score was significantly higher (20.3 ± 2.8) in patients who were implanted with the 2nd-generation PENTA prosthesis in the index operation compared to the mean score (18.5 ± 2.5) of patients implanted with the 1st generation (*p* = 0.005). Patients who underwent primary PENTA procedure with the 2nd-generation implant had the highest mean overall radiographic score of 20.8 ± 2.5 out of 24.

**Fig. 2:**
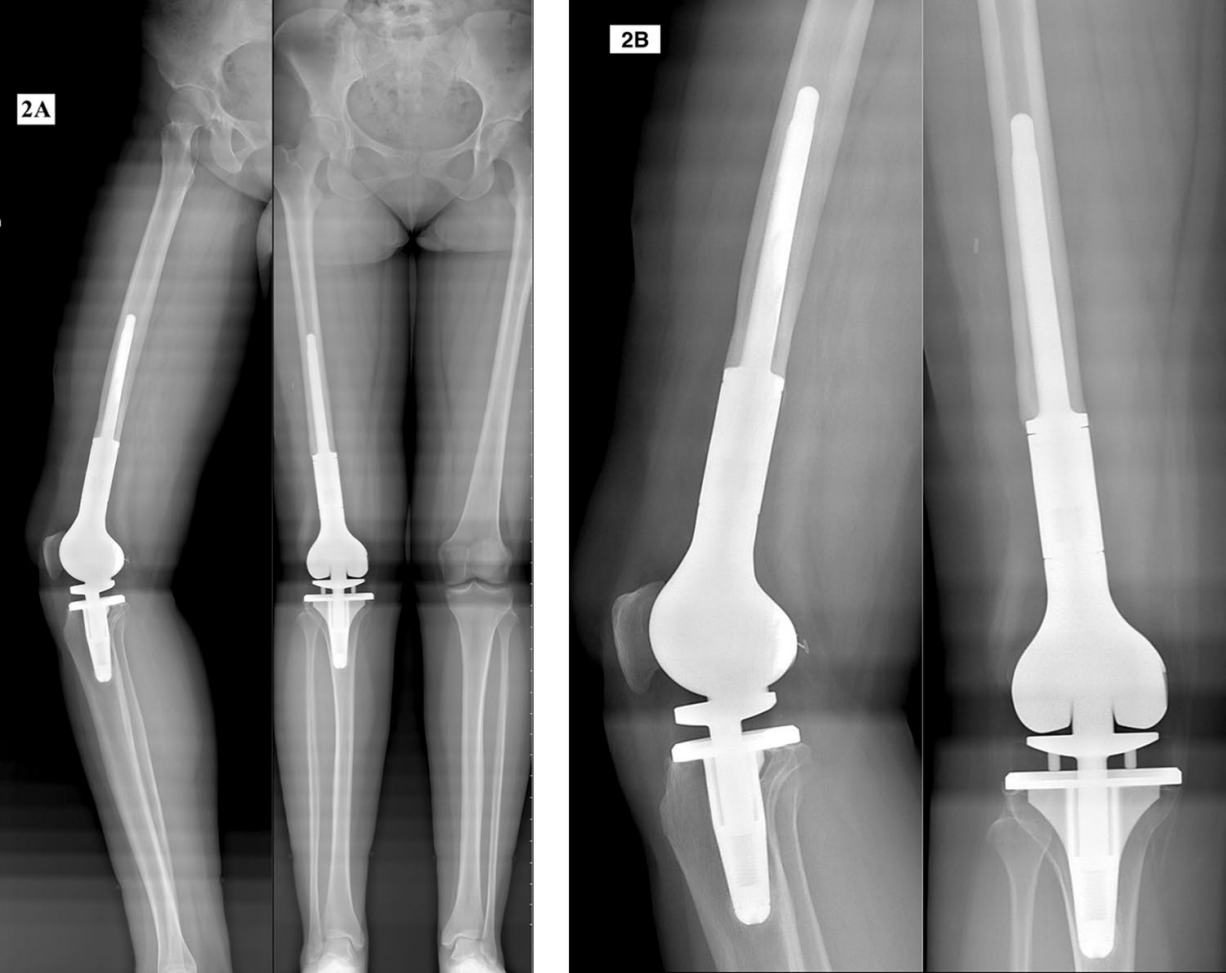
17-year-old female osteosarcoma patient underwent right distal femoral replacement with PENTA® following neoadjuvant chemotherapy. **A** Orthoroentgenograms at 5 years postoperatively. **B** Plain radiographs at the same follow-up demonstrate the bone remodeling around the stem

The mean scores for each radiographic subcategory were as follows: bone remodeling (3.2 ± 1.0). interface (3.4 ± 0.6). anchorage (3.8 ± 0.7). implant body (4.0). implant articulation (3.1 ± 1.3) and extracortical bone bridging (2.2 ± 1.1) out of 4 points. Thus, 5 out of 6 radiographic parameters demonstrated good to excellent outcomes while extracortical bone bridging was fair to good. The mean implant articulation score was significantly higher (3.5 out of 4) with the 2nd-generation implant compared to the mean of 1.8 out of 4 with the 1st-generation implant (*p* = 0.000) (Fig. 2).

### Megaprosthesis complications

*Soft tissue failure, Type 1*: This was observed in 20–22.2% patients. Most of soft tissue failures occurred as in the form of hematoma/seroma formation, wound dehiscence or superficial necrosis. These problems were treated with early aggressive debridement and vacuum-assisted closure techniques. In two patients, hematoma and patella subluxation occurred and were treated by debridement, medial plication and lateral release. In 3 patients, treatment required free flaps. No patient in this group required implant revision attributable to these complications and none of these complications were regarded as having a causal relationship with the use of PENTA implant.

*Aseptic loosening, Type 2*: Aseptic loosening was observed around the femoral stems in 2–2.2% patients who had primarily been treated for osteosarcoma and had undergone revision surgery with PENTA in the setting of aseptic loosening of another implant. Both cases had required extensive bone graft impaction on the femoral side during revision surgery due to loss of bone stock and enlargement of the medullary cavity. Bone remodeling, interface and extracortical bone bridging scores were poor for both patients. Resorption of impacted graft material, thus biological insufficiency, seemed to be the cause of aseptic loosening for these cases.

*Structural failure Type 3*: Structural failure was observed in 29–32.2% patients. A total of 19 hinge failures, 7 peri-prosthetic fractures and 5 anchorage breakages were observed in these 29 patients.

Patients with a poor radiographic "implant articulation" score were uniformly symptomatic and described either mediolateral, posterior or rotationary laxity, clunky sounds and either pain or discomfort in their knee joints. Hinge failure was observed in 13 out of 28–46,4% procedures with the 1st-generation PENTA implant and in 8 out of 73 (11,1%) procedures with the 2nd-generation implant. While hinge failures were significantly more common in proximal tibia cases (*p* = 0.020), no other significant etiologic relationship could be demonstrated with any other demographic data.

Although periprosthetic fracture was encountered in 7 patients, the stem was retained in all cases. The ISOLS radiographic score for "bone remodeling" was recorded as poor "*by definition"* in 5 and as good in 2 out of these 7 patients. On the other hand, according to the UCS classification, 5 were type B1 (fracture around the stem, with the stem stable) and 2 were Type C (fracture in the same bone but away from the implant). Both Type C patients and one patient with type B1 required osteosynthesis while the rest were managed conservatively.

Four broken femoral stems were revised and a tibial baseplate with extension stem awaited revision, while this study was prepared. While the "anchorage" scores were uniformly poor "*by definition"* in these patients, their mean "bone remodeling" and "extracortical bone bridging" scores (1.8 and 1.0 out of 4.0, respectively) were also significantly lower than the mean scores for all patients (3.2 and 2.3 out of 4.0, respectively) (*p* = 0.001, *p* = 0.009). These low radiographic scores were caused by lack of bony contact around the stem under the stem collar. Retrospective analysis of early postoperative radiographs and surgical notes revealed that femoral stems were prematurely fixed inside the medullary canal during insertion with part of the stem shaft and collar left unsupported in 4 patients while the impacted bone grafts were resorbed leaving the tibial baseplate unsupported in the remaining patient. The stem diameter was 11 mm in all broken femoral stems. The breakage occurred at the junction of baseplate and extension stem in the tibial case. The interface scores were good in all indicating that the supported stem segments were well fixed. Although 6 out of 7 mechanical stem failures were seen in distal femur and only 1 in proximal tibia, the correlation was not significant.

*Infection, Type 4*: Peri-prosthetic deep infection was observed in 5–5.6% patients. One patient was treated with debridement, revision of hinge mechanism including polyethylene parts, antibiotic-loaded cement beads and soft tissue reconstruction with latissimus dorsi free flap. Other four patients were treated with intravenous antibiotics, debridement and vacuum-assisted closure. Two of these patients developed chronic fistula and intractable infection. They were then offered trans-femoral amputation but refused amputation. These patients were still ambulatory with the implants clinically and radiologically stable despite ongoing infection at the last follow-up.

*Local recurrence, Type 5*: Local recurrence was observed in 6–6,7% patients (5 malignant, 1 benign). One patient with fibrosarcoma underwent trans-femoral amputation. Five patients were treated with wide re-excision retaining the implants. Four of these 6 patients died with lung metastases.

The failure modes are summarized in Table 2.

**Table 2:** Failure modes and frequencies

Failure mode	Number of patients (%)
Type 1 (soft tissue failure)	20 (22.2)
Type 2 (aseptic loosening)	2 (2.2)
Type 3 (structural failure)	29 (32.2)
Type 4 (infection)	5 (5.6)
Type 5 (local recurrence)	6 (6.7)

### Implant survival analysis

**Fig. 3:**
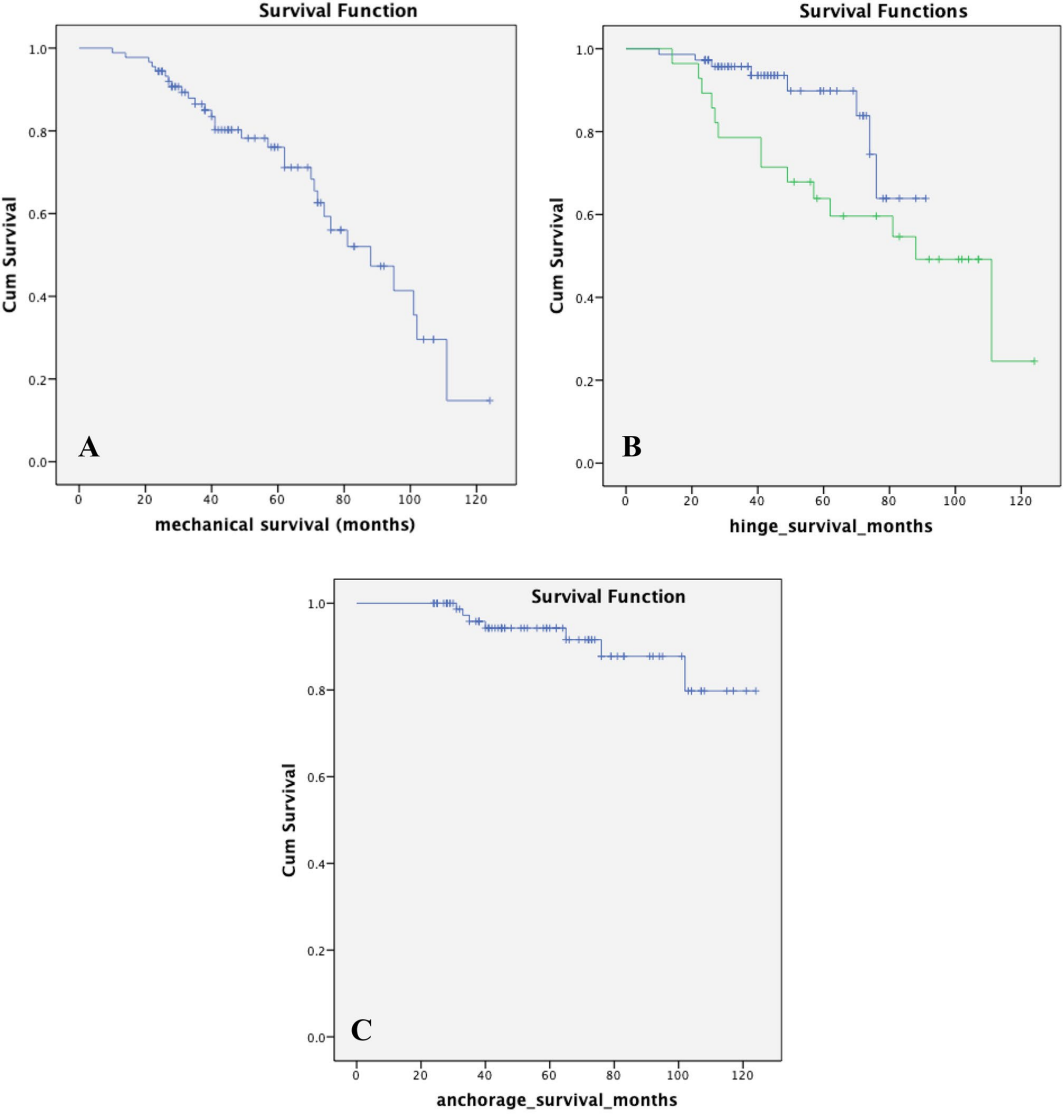
Mid-term Kaplan–Meier survival analyses of the PENTA®. **A** 5-year overall mechanical survival graph. **B** 5-year hinge survival graph (The green and blue lines represent 1st and 2nd generations, respectively). **C** 5-year anchorage survival graph

The overall mechanical 5-year survival rate (including Type 2 & 3 failures) was 81.3% (47 patients) and 92.3% (15 patients) for the 2nd-generation PENTA as primary or revision implant, respectively. On the other hand, 5-year overall mechanical survival rate was 76.1% for all index PENTA operations in 90 patients (Fig. 3A). The 5-year hinge survival rate was 80.4% for all 101 PENTA procedures. However, the 5-year hinge survival rate was 89.8% for procedures with the 2nd-generation hinge mechanism and this was significantly better than 63.9% 5-year hinge survival rate for procedures with the 1st-generation hinge mechanism (*p* = 0.041) (Fig. 3B). The 5-year and 10-year anchorage survival rates were, respectively, 94.3% and 79.8% including all index PENTA operations (Fig. 3C). Anchorage survival rates did not demonstrate significant difference between primary and revision surgeries, 1st and 2nd generations of the implant and whether or not hinge failure occurred.

### Oncological outcome

Distant metastases were observed in 15–16.7% patients and 10–11.1% patients died of disease during follow-up.

## Discussion

As life expectancy and rate of limb salvage in patients with primary malignant bone tumors increase, the durability of the reconstruction becomes more important. Durability of the endo-prostheses is closely related to their design features. While hinge mechanism revisions are more common due to the wear of mobile parts, failure and subsequent revision of anchorage components cause greater morbidity associated with tumor prostheses.

The censored data of excluded patients (30 out of 120 PENTA knee reconstruction patients) might be considered as a limitation of this study. While 4 out of these 30 were operated for non-neoplastic conditions and compromised the homogeneity of the study population, the remaining 26 were excluded either because they died of disease or were lost to follow-up due to logistic and/or socioeconomic reasons, before 2 years. Since the majority of patients undergoing mega-prosthetic knee reconstruction are those with malignant musculoskeletal tumors, limited follow-up due to death is a natural occurrence in such a cohort. While the insufficient data of patients, which were left out, might decrease the statistical power of the study, we believe that the outcome analyses are not skewed by our exclusion criteria. Revision PENTA procedures as well as primary procedures were included in the study since all revision procedures were performed on patients, who had also initially undergone limb salvage for oncological reasons and endo-prosthetic interventions are afflicted by common problems, such as previous oncological treatment, defective and weakened bone stock, weak soft tissue coverage, and extensive muscle loss in both groups.

A brief review of global mega-prostheses used over the last decades yields several major designs and implant systems (Table 3). In Kotz Modular Femur-Tibia Reconstruction System (KMFTR), diaphyseal fixation was obtained with screws going through the 2 lateral flanges [12]. This design usually failed through progressive wear of the polyethylene and loosening, and stem breakage at the site of the nearest screw hole [13–15]. Howmedica Modular Reconstruction System (HMRS) had an improved design over KMFTR and had a single flange to reduce stress shielding [13, 16, 17]. Ruggieri et al. evaluated both of these fixed hinge prostheses [18] and showed 4.8% breakage in prostheses requiring revision (10,5% in KMFTR, 3,5% in HMRS). HMRS prostheses had a significantly lower rate of breakage compared to KMFTR. Aseptic loosening rates were not found to be significantly different (4.9% in HMRS, 9.6% in KMFTR). Global Modular Reconstruction System (GMRS) is an improvement on HMRS with its rotating hinge. Rotating hinge decreased mechanical stresses and complications at the bone–implant interface. Aseptic loosening rates (5%), although decreased with the introduction of rotating hinges remain a common cause implant failure [4, 18].

**Table 3:** Outcomes and survival in major distal femoral and proximal tibial megaprosthetic reconstruction series

Literature	Site, brand	Follow-up (m)	*N*	Type 1 (*n*, %)	Type 2 (*n*, %)	Type 3 (*n*, %)	Type 4 (*n*, %)	Type 5 (*n*, %)	5-year implant survival (%)
Pala et al. [4]	DF (GMRS)	48 (24–96)	187	13 (6.9)	10 (5.3)	0	16 (8.6)	11 (5.9)	
	PT (GMRS)	48 (24–96)	60	8 (13.3)	4 (6.7)	0	7 (11.7)	3 (5.0)	
Gosheger et al. [6]	DF (Mutars)	45 (3–140)	103	–	15 (14.6)	3 (2.8)	12 (11.7)	–	66
	PT (Mutars)		42	–	3 (7.1)	1 (2.4)	7 (16.7)	–	62
Ruggieri et al. [18]	HMRS	132 (24–300)	543		26 (4.9)	19 (3.5)	45 (8.4)		80 (10 yr)
	KMFTR		126		12 (9.6)				
Heisel et al. [24]	Mutars	46 (24–84)	50	–	11 (22)	5 (10)	6 (12)	–	–
Capanna et al. [29]	DF (megasystem)	67 (24–149)	87	4 (4.6)	3 (3.4)	18 (20.7)	12 (13.8)	4 (4.6)	70
	PT (megasystem)	67 (24–149)	32	3 (9.4)	0	0	3 (9.4)	2 (2.3)	84
Myers et al. [30]*	DF (Stanmore)	144 (60–360)	173		41 (24)	–	32^**^(9.6)	–	83^**^
Myers et al. [31]*	PT (Stanmore)	176 (60–348)	99		3 (3)		37^&^ (19.5)		68^¶^
Current study	DF (PENTA)	60 (24–121)	56	13 (23.2)	1 (1.8)	15 (26.8)	3 (5.4)	6 (10.7)	91
	PT (PENTA)	56 (24–124)	34	7 (20.6)	1 (2.9)	14 (41.2)	2 (5.9)	0	100

*DF* distal femur, *PT* Proximal tibia

^*^Only rotating hinge group is depicted

^**^Out of 335 patients

^¶^Out of 194 patients

Dynamic compression fixation, the philosophy behind Compress Pre-Stress Implant (Biomet Inc, Warsaw, IN), was a novel idea to reduce osteolysis in the proximal tibia and distal femur. These stems provided stem stabilization without the need for long stems, decreased stress shielding and osteolysis and increased osteointegration. Most common complications of this implant were aseptic loosening (9.7%) and fracture of the underlying bone between the anchor plug and the spindle [19, 20].

While cement-less stems offer the crucial advantage of osteointegration and are therefore expected to yield better survival outcomes with regard to aseptic loosening, some authors advocate the use of cemented prostheses mainly due to better stress-shielding properties. The survival outcomes of Stanmore prostheses with cemented stems have been studied extensively over long follow-up periods in large study populations. Unwin et al. reported aseptic loosening as the most common cause of failure with 10-year stem survival rates of 67.4% and 54.8%, respectively, in distal femur and proximal tibia in a series with 1001 patients. Coathhup et al. on the other hand, reported significantly improved 10-year survival of 88.9% against aseptic loosening with the use of HA-coated collars on cemented stems in Stanmore distal femur prostheses [21–23].

MUTARS (Implancast, Germany) was introduced to the market in 1995. It can be inserted cemented or press-fit and has a hexagonal cross section. This hexagonal design contributed to good rotational stability and low rates of aseptic loosening (8%) according to a 250 patient series by Gosheger et al. [6]. The deep infection rate was found to be 12% and stem breakage rate was 1.6% in the same study. Another study on 100 patients by Heisel et al. showed an aseptic loosening rate of 22% and deep infection rate of 12% [24].

The aseptic loosening rate of 2.2% in our study series was remarkably lower compared to other established prosthesis designs. loosening rate was 2.2%. This outcome can be attributed to HA-coated pentagonal, slightly tapered and collared stems, which provide exceptional primary and secondary stability. Radiological criteria support this successful outcome with regard to anchorage, bone remodeling and bone–implant interface scores.

Although no clear support in the literature exists regarding cemented and cement-less fixation, some authors suggested that cement-less fixation might be advantageous due to bone integration and lower aseptic loosening rates. To increase osteointegration between implant and the surrounding bone, cement-less press-fit stems have been constructed in different shapes (fluted, fenestrated) and different textures (grit-blasted, porous-coated, beaded, hydroxy-apatite coated). In their 232 patient study, Pala et al. compared cemented and cement-less fixation and observed cement-less fixation group to have higher overall survival and survival to infection whereas survival to aseptic loosening was not significantly different than the cemented group [25]. Cemented fixation may be appropriate in patient with bone metastases, extensive osteolytic defects, bad prognosis and elderly patients. In young patients with primary bone tumors, cement-less fixation is preferable [14, 25]. In this study un-cemented PENTA stems resulted in excellent midterm survival.

To achieve the best results, the prosthesis must respect the biomechanics. Early tumor prosthesis designs with fixed hinges failed due to torsional forces on the anchorage sites, which resulted in aseptic loosening. Newer designs with rotating hinges tried to emulate the physiological rotation of the knee joint through the arc of motion. These rotating hinge designs also had their share of problems. This sudden stop in some cases resulted in loosening around the junction of stem and body, which further led to implant breakage and peri-prosthetic fracture. We have encountered such cases in designs with a single canal in polyethylene for rotation. Another possible design for rotating hinges uses elevation of the plateau during rotation as a screw-home mechanism; however, if prosthesis continues to rotate, dislocation can occur. PENTA has two canals in the polyethylene insert for rotation. This allows for load distribution at the ends of rotation. The PE insert of PENTA has a concave surface to improve contact surface with femoral condyles and increase stability with a smooth stop.

Implant failure includes both stem revision and revision of joint mechanism. First-generation PENTA designs had issues with early wear of the bumper in hyperextension. The wear of the bumper was followed by metal abrasion and cracking or wear of the axle bushing, which eventually led to global instability. The sharp edge of the femoral notch, which damaged the bumper insert was beveled and hyperextension loads were also distributed through stepped rotation blocks on the axle head and inside the axle socket of the femoral component with a design change. High body mass index and excessive hyperextension loading during ambulation due to extensor muscle loss may have contributed to the wear of the polyethylene bumper in these patients.

Peri-prosthetic deep infection is a major mode of failure in patients with knee mega-prostheses. Coating implant with silver was introduced as a sophisticated and promising strategy against this complication in 2000s. Successful prevention and treatment of peri-megaprosthetic joint infections in oncological patients undergoing surgery for different anatomic locations have been reported in both original studies and meta-analyses [26, 27]. While the infection rate for patients undergoing primary megaprosthetic reconstruction with silver-coated implants ranged from 9 to 10%, a significantly reduced re-infection rate of 13.7–29.2% was reported when silver-coated implants were used instead of uncoated implants in revision surgery [26, 27]. On the other hand, another review of the literature by authors, who have long advocated and have substantial experience with the use of silver-coated implants, emphasizes that long-term definitive evidence is needed to show effectiveness of silver and it is only expected to prevent biofilm formation [28]. Deep infection rate was remarkably low in our study population when compared with infection rates from the literature (Table 3). Although this excellent outcome might be attributed to the PENTA implant, periprosthetic infection is obviously multifactorial and cannot be evaluated independent from variables like surgical technique and management of soft tissues.

## Conclusion

PENTA megaprosthesis system is a reliable choice for the reconstruction of tumor-related massive osteoarticular defects around the knee. The results demonstrate that the design issue of the 1st-generation hinge was resolved in the 2nd generation. Although long-term follow-up is necessary for a definitive evaluation of the implant's survival characteristics and performance, short to mid-term follow-up yields exceptional anchorage properties with very good functional outcomes.
